# Supplementary material for: Enhanced metabolic entanglement emerges during the evolution of an interkingdom microbial community
Source: Nat Commun. 2024 Aug 22;15:7238. doi: 10.1038/s41467-024-51702-1 (PMC11341674; doi:10.1038/s41467-024-51702-1)
Supplement: Supplementary file 1 — Supplementary Information [file 41467_2024_51702_MOESM1_ESM.pdf]

Supplementary Information

Enhanced metabolic entanglement emerges during the evolution of an interkingdom microbial community

Giovanni Scarinci[1,2], Jan-Luca Ariens[1,2], Georgia Angelidou[1], Sebastian Schmidt[1,2], Timo Glatter[1], Nicole Paczia[1], Victor Sourjik[1,2]*

[1]Max Planck Institute for Terrestrial Microbiology, Marburg, Germany

[2]Center for Synthetic Microbiology (SYNMIKRO), Marburg, Germany

* corresponding author (victor.sourjik@mpi-marburg.mpg.de)

Content of Supplementary information

1. Supplementary Figures 1-10
2. Supplementary Tables 1-3

Supplementary Figures

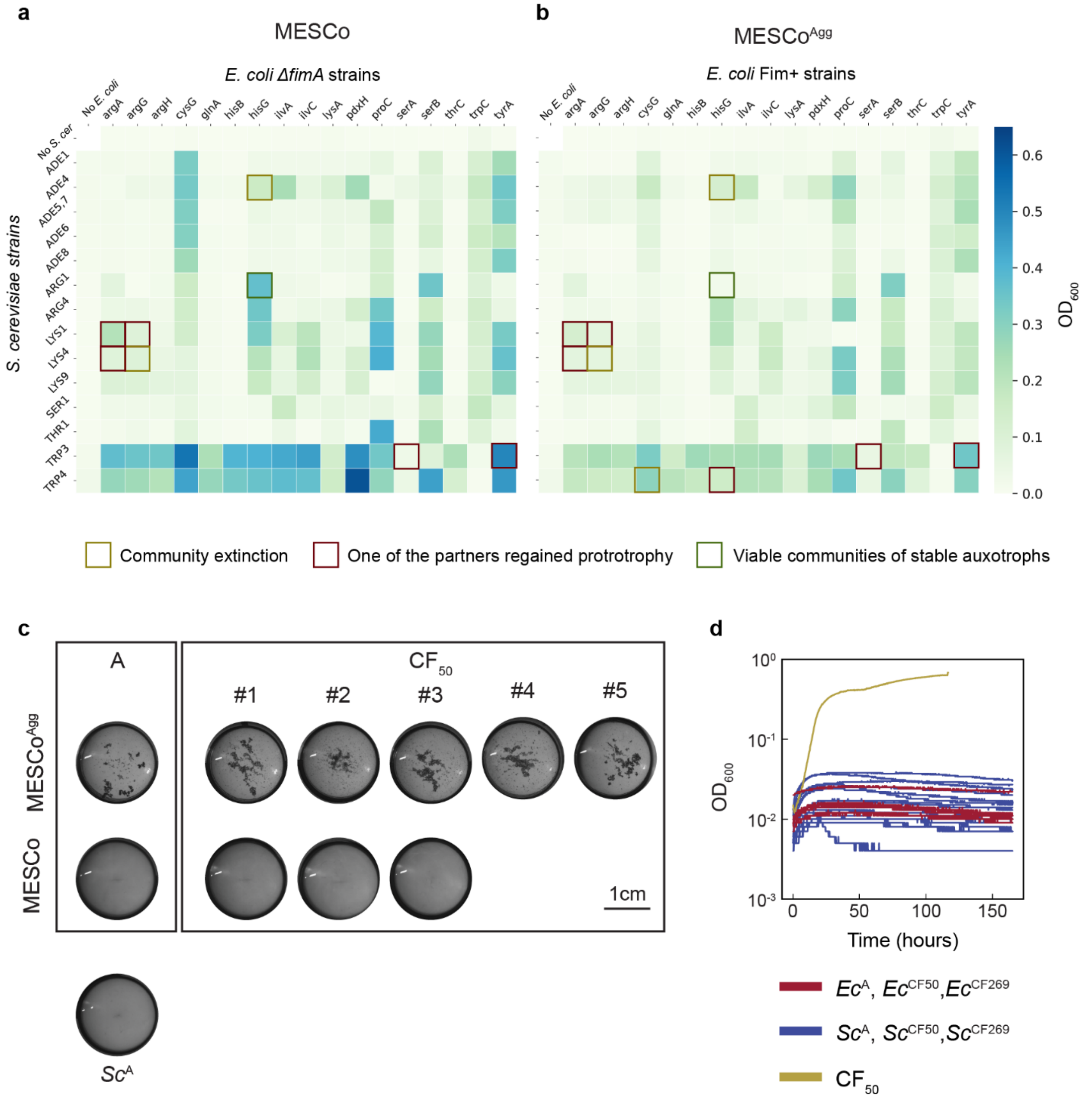

Supplementary Figure 1. Design and evolution of MESCo communities

(**a**,**b**) Growth of pairwise co-cultures between different *E. coli* and *S. cerevisiae* strains carrying indicated deletions of metabolic genes, assembled using either fimbrialess (*ΔfimA*; a) or fimbriated (Fim^{+}; b) *E. coli* partner strains. Color scale indicates density of the co-culture (OD_{600}) after 120 h of cultivation at 30 °C and 200 r.p.m. in CF-MM. A subset of these communities (highlighted squares) have been co-cultured over multiple passages (between 10 and 15) to assess for community stability. These were selected to cover different ranges of final OD600 and also to cover communities carrying deletions in the same pathway but at different positions. Different colors of the square frames indicate whether a community was stable or become extinct, and whether the auxotrophy was retained, for which individual partners were isolated from the co-culture and assessed for their growth as a monoculture in CF-MM. (**c**) Aggregation test for the non-aggregative (MESCo)

and the aggregating ($MESCo^{Agg}$) communities, either ancestral (A) or after co-culture evolution for 50 generations in CF-MM (CF_{50}). To test their aggregation, the communities were incubated in PBS for with shaking at 200 r.p.m. for 1 h at 30 °C in a 24-well plate (Greiner Bio-One GmbH). Also shown is the control culture of the ancestral yeast partner (Sc^A) alone. (**d**) Growth, measured as OD_{600} using a plate reader, of monocultures of indicated strains in CF-MM.

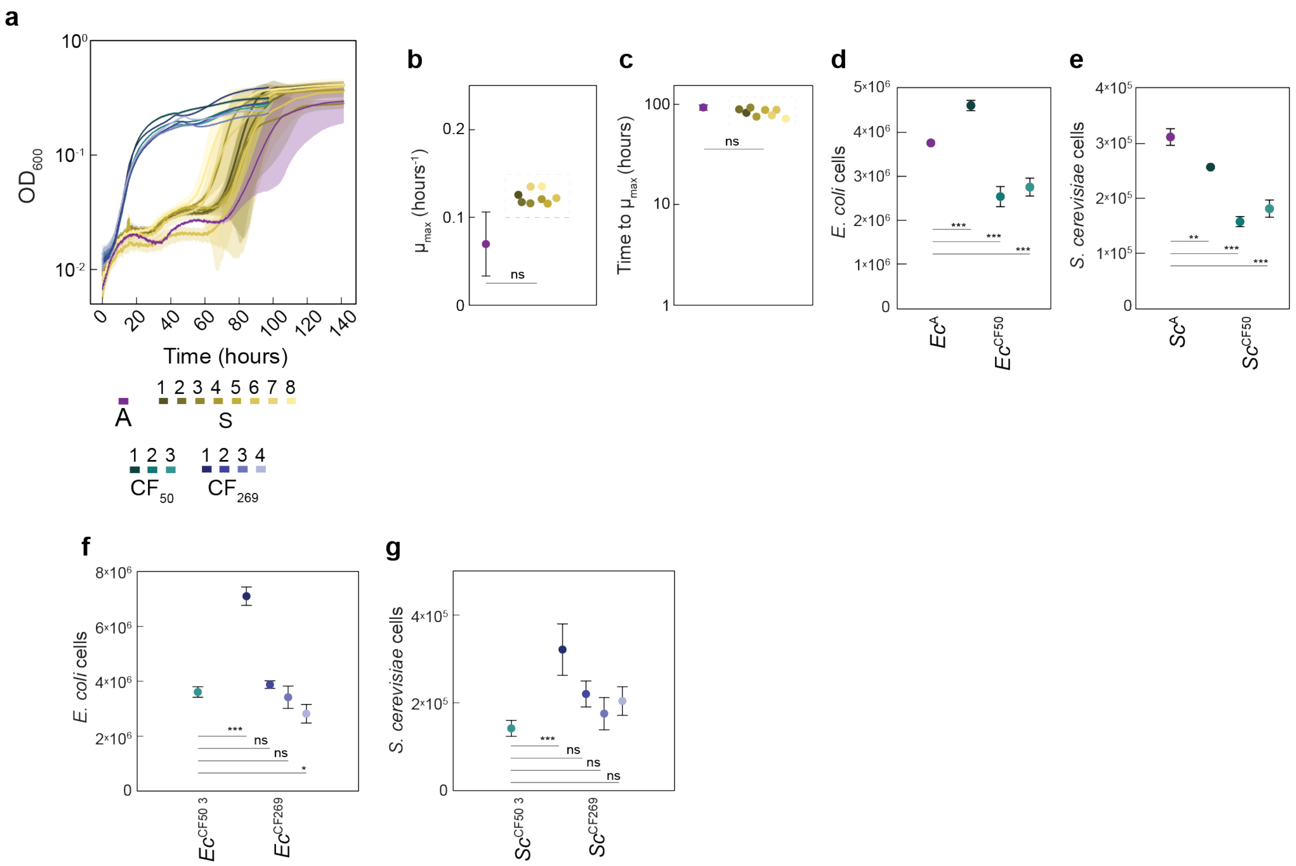

Supplementary Figure 2. Characterization of growth parameters of the evolved communities

(**a**) Growth profiles of all the eight MESCo communities evolved in AH-MM compared to communities shown in **Figure 1b**. (**b,c**) Maximum growth rate (b) and time to reach it (c) for the ancestral MESCo (A) and the MESCo evolved in AH-MM (S), calculated from the curves shown in (a). The value for A represents the mean from three independent cultures, with the error bar indicating SD. Different colors used for S represent different individual evolved cultures. (**d,e**) Cell counts of *E. coli* (d) and *S. cerevisiae* (e) measured using flow cytometry at the final time point (144 h) of the curves shown in **Figure 1c**. The different colors used for the evolved organisms indicate individual evolved lines. Mean values of n = 3 biological replicates ± SD are shown. (**f,g**) Cell counts of *E. coli* (f) and *S. cerevisiae* (g) for the indicated MESCo communities, measured at the final time point of the curves shown **Figure 1b**. Different colors for the evolved organisms indicate individual evolved lines. Mean of n = 3 biological replicates ± SD. *p* values (ns = $p > 0.05$, * = $p < 0.05$, ** = $p < 0.01$, *** = $p < 0.001$) reported in (b) and (c) are from a two tailed *t*-test assuming unequal variance between the samples while in (d,e,f,g) from a one-way ANOVA followed by Tukey *post-hoc* test.

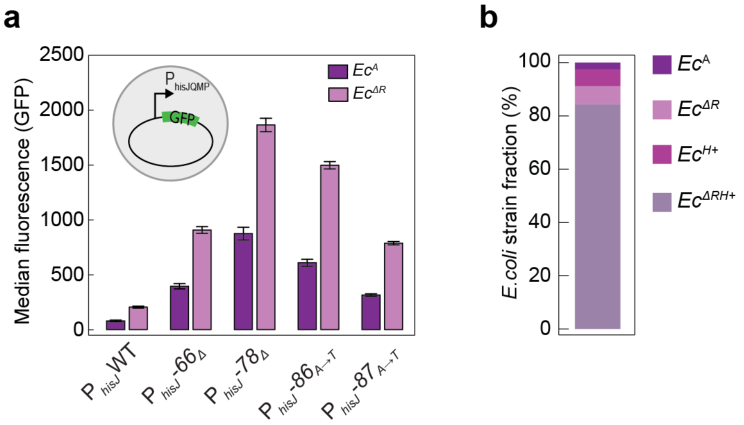

Supplementary Figure 3. Impact of mutations on *E. coli* and on its fitness

(**a**) Median fluorescence intensity of GFP reporter plasmid, measured using flow cytometry for reporters carrying point mutations identified in the promoter of the *hisJQMP* operon in evolved *E. coli* lines. Reporter plasmids were transformed into ancestral (Ec^{A}) or *ΔargR* mutant ($Ec^{\Delta R}$) *E.coli* strain, as indicated, and cultures were grown in CF-MM supplemented with 20 mg/l histidine for 45 h. Mean values of $n = 3$ biological replicates ± SD are shown. (**b**) Average final fraction of indicated *E. coli* strains, initially co-inoculated at the same initial density ($OD_{600} = 0.0125$ each) together with the ancestral *S. cerevisiae* strain, and grown in CF-MM for 72h**.** Mean values of n = 6 biological replicates are shown, with SD (not shown) below 1%.

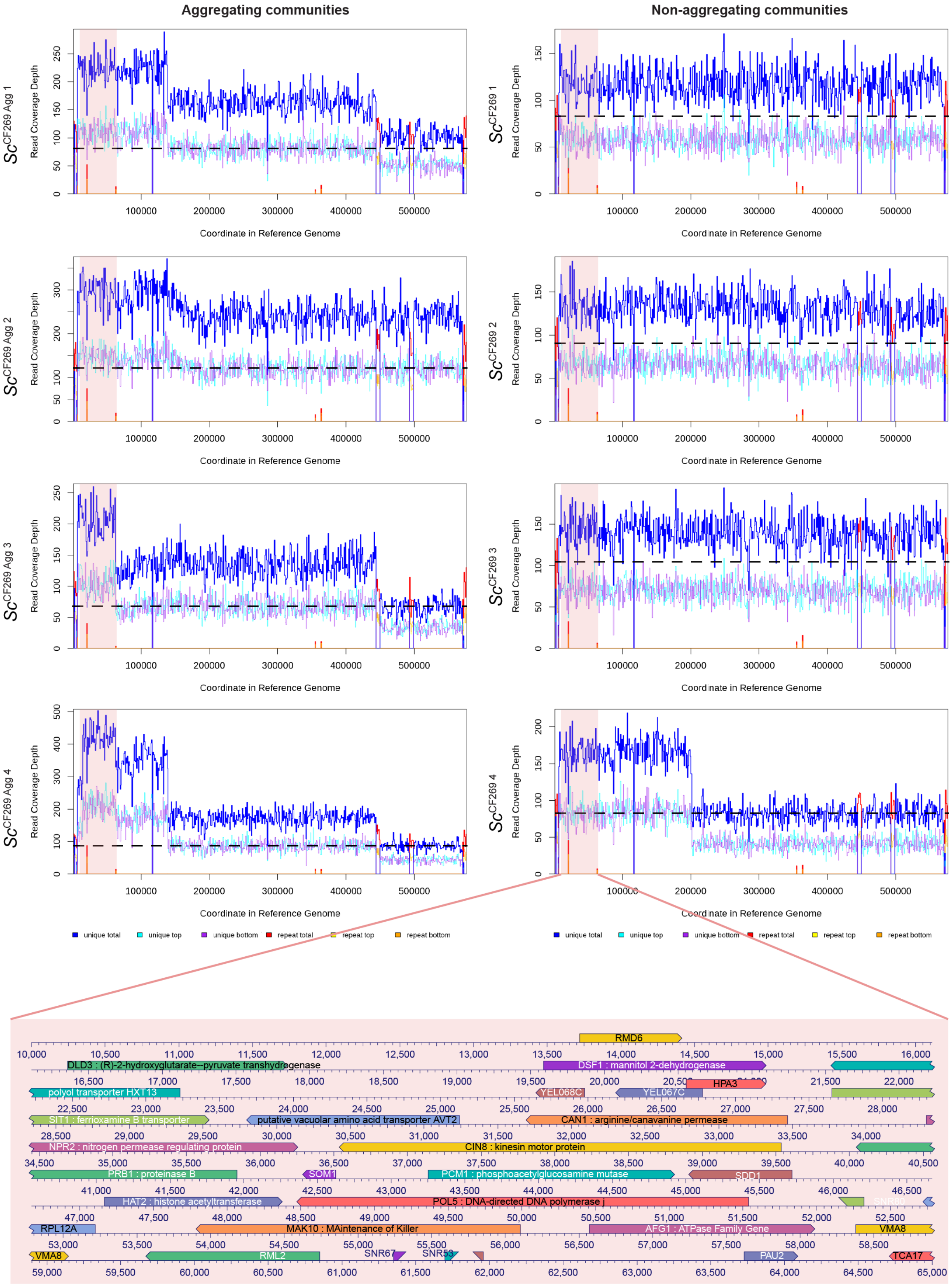

Supplementary Figure 4. Duplications and aneuploidies in chromosome V in the evolved yeast lines

Read coverage depth obtained in Illumina sequencing on chromosome V in yeast lines in aggregating (left) or non-aggregating (right) MESCo communities evolved for 269 generations in CF-MM. Light blue and purple lines represent the read coverage from paired-end reads matching only once in the reference genome, with the dark blue line indicating their sum. Yellow and orange lines represent the read coverage from pair ends reads matching more than once in the reference genome (e.g. repetitions) and normalized by the number of repetitive sequences found in the genome, with the red line representing their sum. The pink area represents the chromosomal region between nucleotides 10 000 and 60 000 that underwent repetitive events of duplications

during evolution, with genes present in this region shown in the area. The dashed black line indicates the mean total read coverage depth for the other chromosomes in each yeast line.

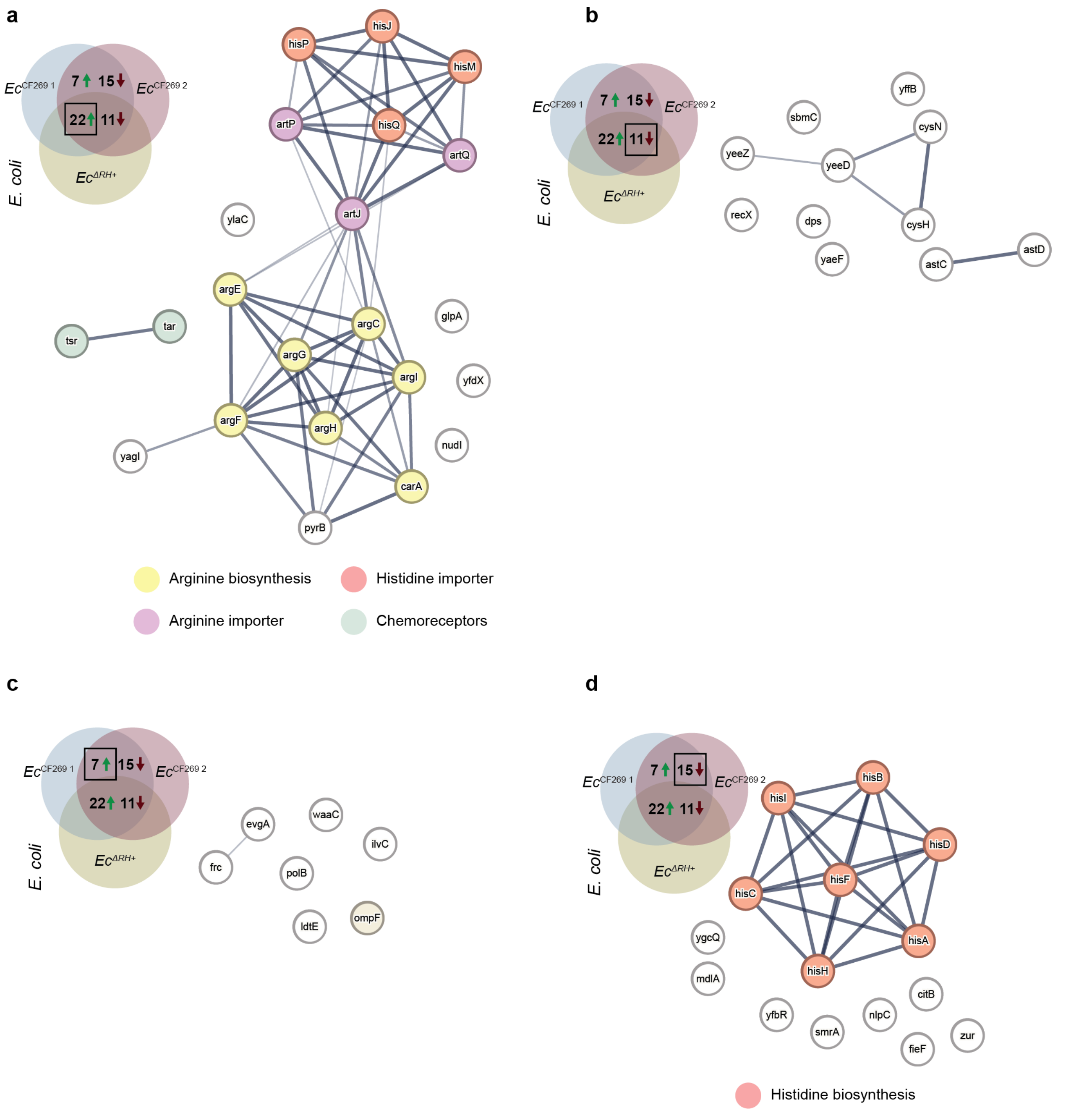

Supplementary Figure 5. Proteins with different expression levels between indicated *E. coli* strains and the ancestral strain

(**a-d**) STRING[64] analysis depicting proteins that are upregulated (a,c) (log2(FC) > 1, -Log(p) > 1.5) or downregulated (b,d) (log2(FC) < 1, -Log(p) > 1.5) either in both the mutant and two of the evolved *E. coli* (a,b) or only in the evolved *E. coli* lines (c,d) compared to the ancestral strain. Highlighted are clusters of proteins sharing common functions. Comparison was performed between the evolved communities, the reconstituted communities of mutants carrying major mutations, and the ancestral community. Because of differences in growth between the ancestral and the evolved or mutant communities, only proteins with different expression levels at both 36 h and 100 h were selected. FC = Fold change in total protein intensities.

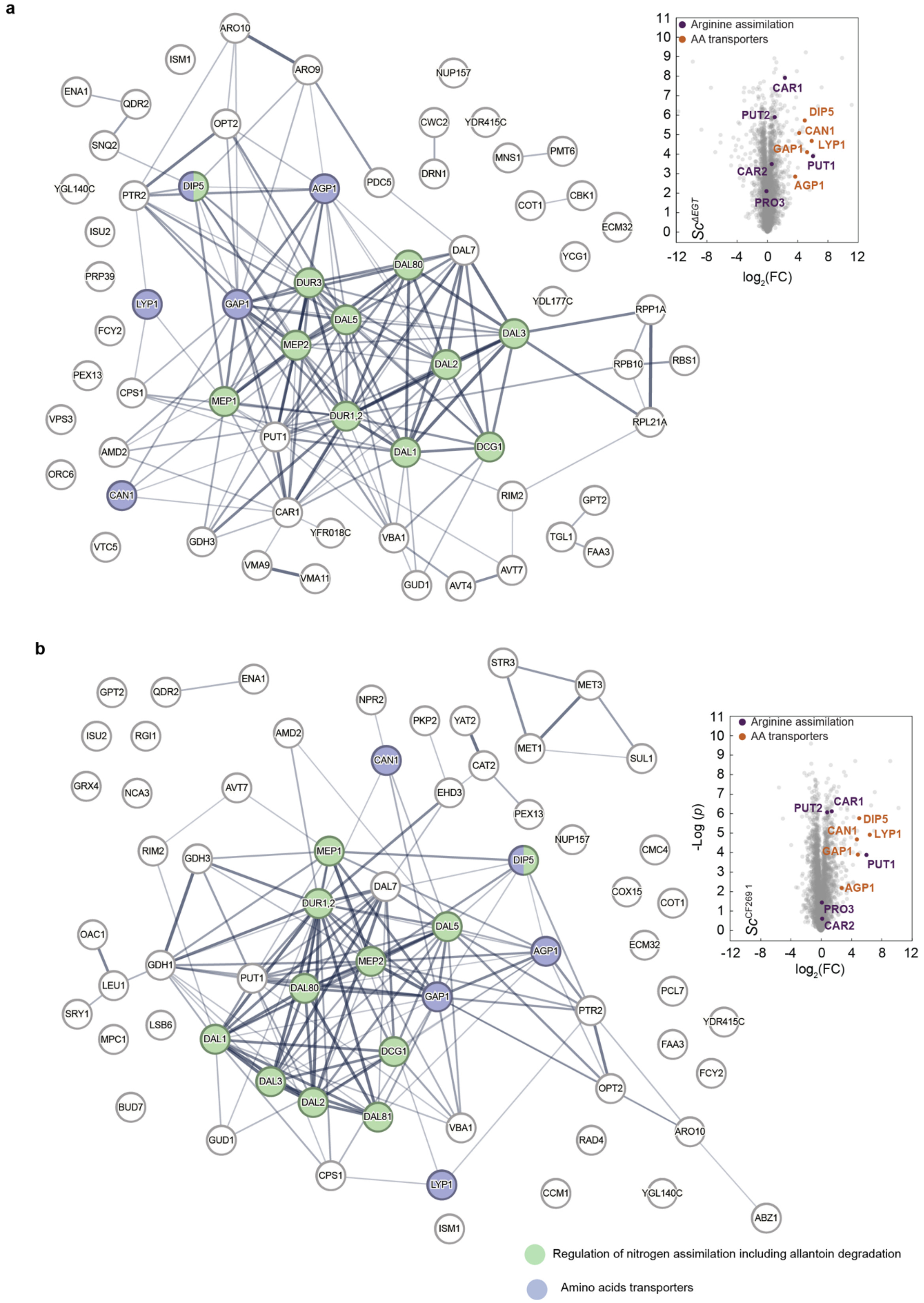

Supplementary Figure 6. Proteins upregulated in different *S. cereevisiae* lines compared to the ancestral strain in co-cultures with *E. coli ΔargR*

(**a,b**) Volcano plots representing the proteome difference from the ancestral strain and STRING[64] analysis depicting proteins upregulated (log2(FC) > 2, -Log(p) > 2) compared to the ancestral yeast strain in the $Sc^{\Delta EGT}$ mutant (a) and in one of the evolved yeast line ($Sc^{269\ 1}$) (b). All strains were co-cultured with the $Ec^{\Delta R}$ mutant strain for 36 h in CF-MM. For STRING analysis, highlighted are the clusters of proteins involved in either

amino acids uptake (blue) or in the regulation of nitrogen utilization and allantoin degradation (green). In volcano plots, the enzymes involved in direct ammonium assimilation (purple) and the amino acid transporters regulated by *ecm21* (orange) are highlighted.

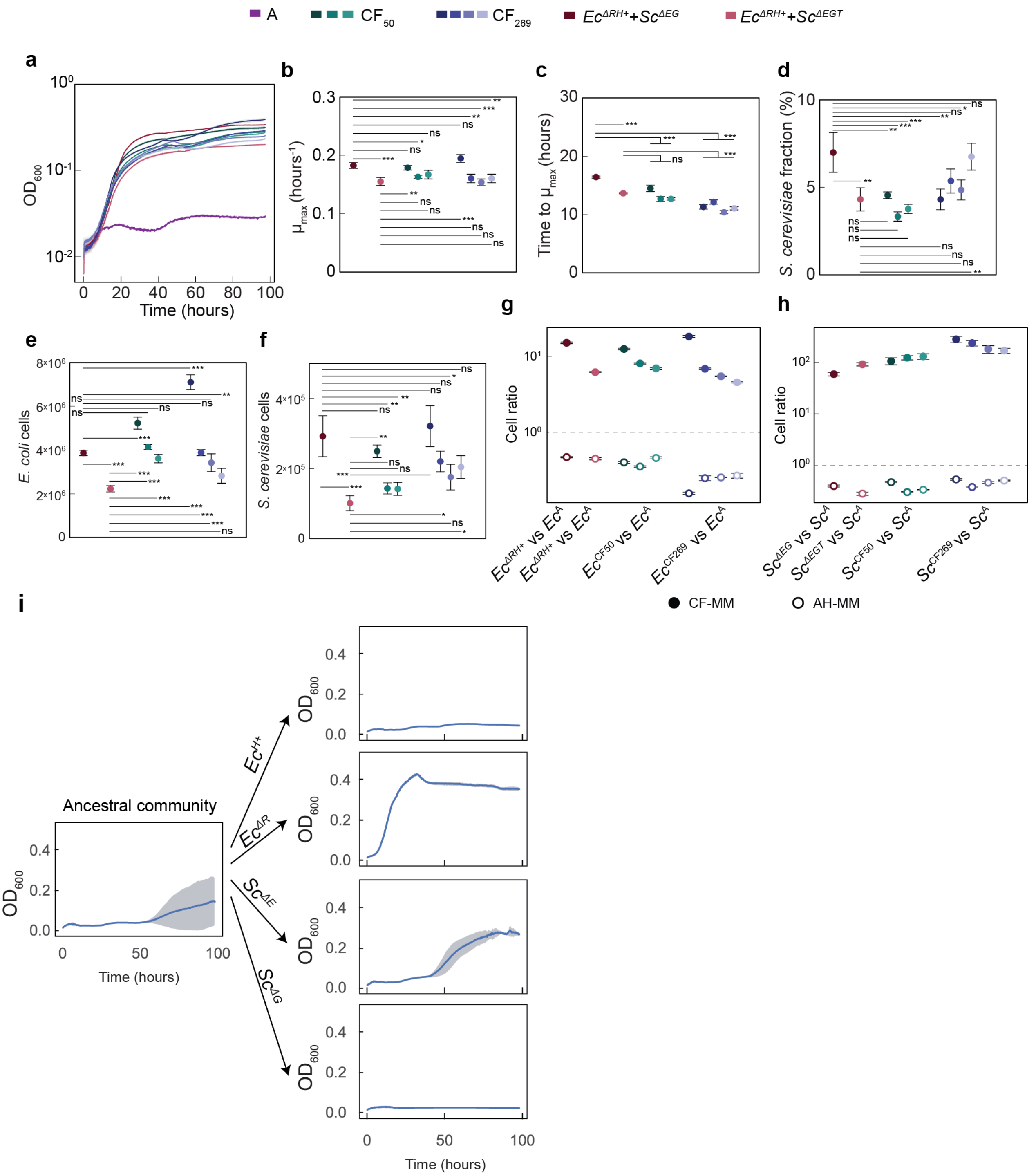

Supplementary Figure 7. Assessing how well communities of mutants recapitulate the growth parameters of evolved communities

(**a**) Growth of communities of ancestral and mutant *E. coli* and *S. cerevisiae* strains in CF-MM, in comparison with the lines evolved for 50 and 269 generations, as shown in **Figure 1b**. Mean values of n = 3 biological replicates ± SD are shown. (**b-f**) Maximum growth rate (b), time to reach it (c), yeast cell fraction (d), the *E. coli* cell count (e) and the yeast cell count (f) from communities shown in (a) and in **Figure 1b**. Mean values of n = 3 biological replicates ± SD are shown. (**g,h**) Cell ratios to the ancestral strain calculated as in **Figure 1g,h**, either in CF-MM or in AH-MM, respectively, for the bacterium (g) or the yeast (h) mutants, in comparison with the evolved strains shown in **Figure 1g,h**. Mean values of n = 5 biological replicates ± SD are shown. (**i**) Growth of the community assembled with the ancestral strains, compared to the communities in which one of the organisms is replaced by a strain carrying one of the high-frequency mutations. Mean values

of n = 3 biological replicates ± SD are shown. *p* values (ns = $p > 0.05$, * = $p < 0.05$, ** = $p < 0.01$, *** = $p < 0.001$) are from a one-way ANOVA followed by Tukey post-hoc test.

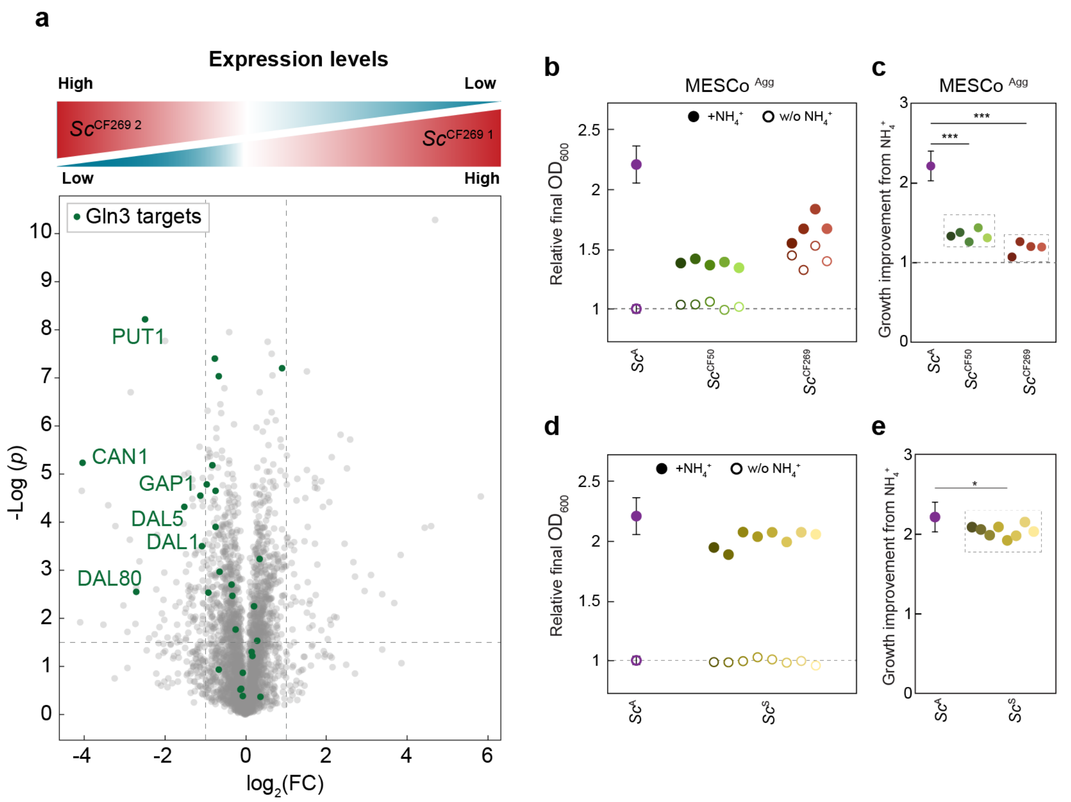

Supplementary Figure 8. Impact of Gln3 truncation on expression of its targets and effect of ammonium on growth of communities evolved under aggregation or in presence of supplements

(**a**) Volcano plot comparing proteome of the two evolved yeast lines, *Sc*$^{CF269\ 1}$ and *Sc*$^{CF269\ 2}$ grown in CF-MM for 36 h. *Sc*$^{CF269\ 2}$ carries mutation leading to expression of the truncated version of Gln3, whereas *Sc*$^{CF269\ 1}$ has the full-length variant of Gln3. Mean values of $n = 4$ biological replicates are shown. Known targets of Gln3 are highlighted in green. (**b,c**) Final OD_{600} of the yeast lines originating from evolved MESCo^{Agg} communities, grown in CF-MM with arginine and either with or without ammonium, relative to the final OD_{600} of ancestral yeast strain grown in absence of ammonium. (d,e) Relative final OD_{600} for yeast strains isolated from MESCo communities evolved for 100 generations in AH-MM and grown in CF-MM with arginine either with or without ammonium. In each panel, values represent the average from two biological replicates, except for the Sc^{A} (same as **Figure 3d**) where 11 biological replicates where averaged. Error bars for Sc^{A} represent SD. *p* values (ns = $p > 0.05$, * = $p < 0.05$, ** = $p < 0.01$, *** = $p < 0.001$) are in (c) from a one-way ANOVA followed by Tukey *post-hoc* test while in (e) are from a two tailed *t*-test assuming unequal variance between the samples.

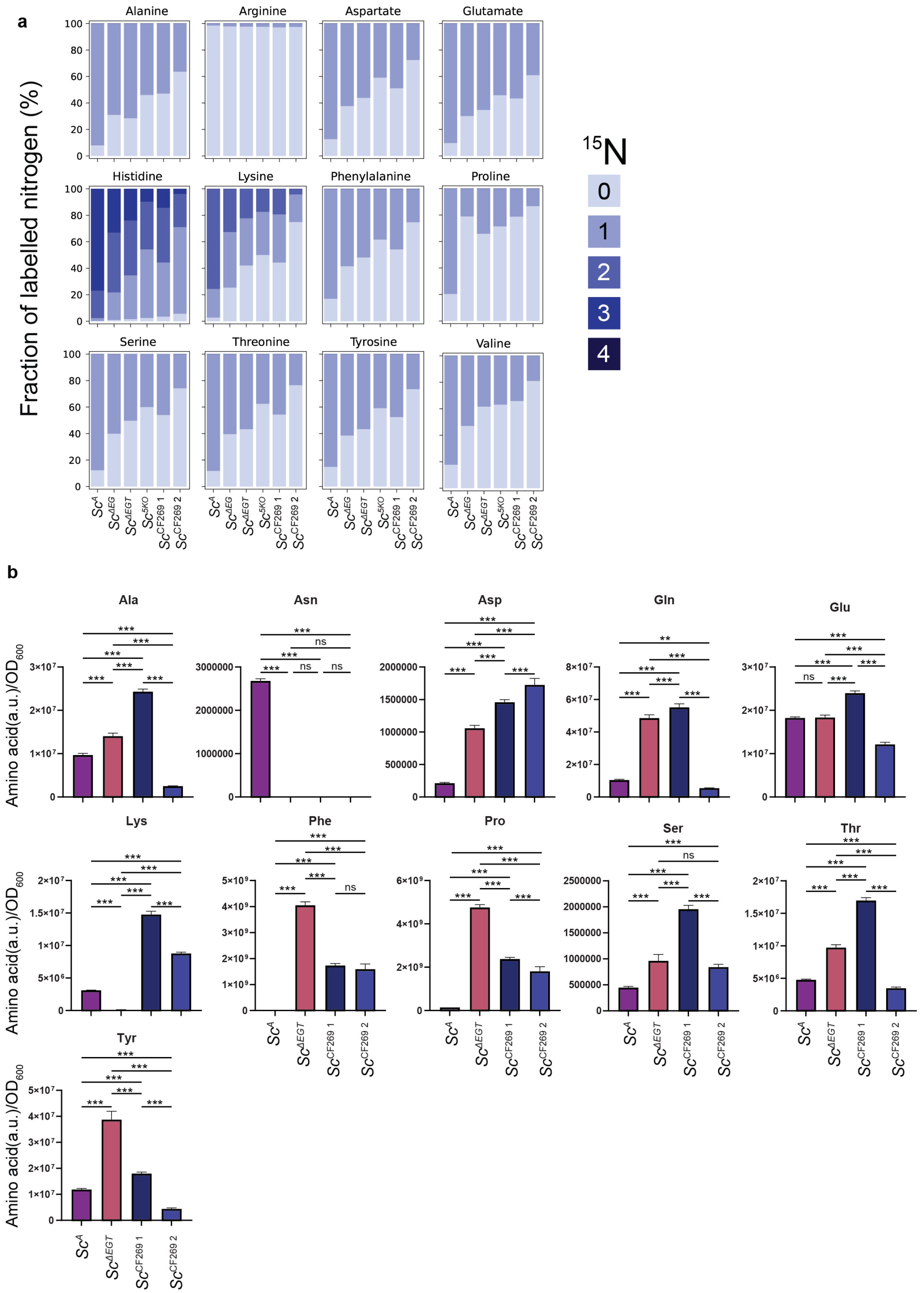

Supplementary Figure 9. Isotope labeling patters and amino acids exometabolome profiles in indicated yeast strains

(**a**) Average fraction of ^{15}N-labelled atoms detected for the full set of proteinogenic amino acids measured in samples described in **Figure 4e,f**. Mean values of n = 4 biological replicates are shown, with SD values (not shown) below 3% for all samples except alanine (4%) and valine (13%) for the $Sc^{\Delta EG}$ strain. (**b**) Levels of indicated amino acids expressed in arbitrary units (a.u.), measured as area under the peak from LC-MS

measurements of the culture supernatant and normalized to OD_{600}. Cultures were the same as in **Figure 4f** and (a). Mean values of $n = 4$ biological replicates ± SD are shown. p values (ns = $p > 0.05$, * = $p < 0.05$, ** = $p < 0.01$, *** = $p < 0.001$) are from a one-way ANOVA followed by Tukey post-hoc test.

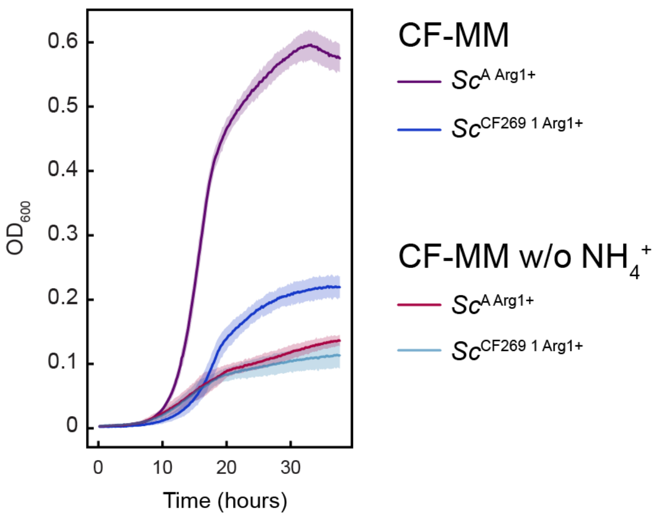

Supplementary Figure 10. Growth of *S. cerevisiae* in CF-MM with or without ammonium

The ancestral and one of the evolved yeast strains with restored arginine prototrophy grown in CF-MM with (data as in Fig.4k) or without ammonium. Mean values of $n = 3$ biological replicates ± SD are shown.

Supplementary Tables

Supplementary Table 1. Statistical analysis **Figure 2i**. Compact Letter Display (CLD) from the Tukey test performed as post-hoc analysis. Strains with the same letter have no statistical difference in intensity levels for the indicated protein. The *p* values from the ANOVA analysis are also reported.

	**Strains**				
**Protein**	Sc^{A}	$Sc^{\Delta EG}$	$Sc^{\Delta EGT}$	$Sc^{CF269\ 1}$	**ANOVA p value**
**PUT2**	a	b	c	d	p<0.001
**PUT1**	a	b	c	c	p<0.001
**CAR2**	a	b	b	a	p<0.001
**CAR1**	a	b	c	d	p<0.001
**PRO3**	a	a	b	c	p<0.001
**CAN1**	a	b	c	c	p<0.001
**GAP1**	a	b	c	c	p<0.001
**AGP1**	a	a	b	b	p<0.001
**DIP5**	a	b	c	c	p<0.001
**LYP1**	a	b,c	cd	d	p<0.001

Supplementary Table 2. Plasmids used in this study

Plasmids	Addgene catalogue number	Reference
pOB2		[66]
pNB1		[67]
pUA66		[68]
pSIJ8	#68122	[52]
pPL5071	#60930	[69]
pH3FS	#85780	[70]
pBAD-lssmOrange	#37129	[71]
pKD45		J. S. Parkinson, personal gift
pGS62 (pTrc99A:mNeonGreen)		This work
pGS63 (pTrc99A:mCherry)		This work
pGS64 (pTrc99A:mTurquoise2)		This work
pGS65 (pTrc99A:lss-mOrange)		This work
pGS66 (pUA66 P_{hisJ} -86$_{A\text{->}T}$:GFP)		This work
pGS67 (pUA66 P_{hisJ} -87$_{A\text{->}T}$:GFP)		This work
pGS68 (pUA66 P_{hisJ} -78Δ:GFP)		This work
pGS69 (pUA66 P_{hisJ} -66Δ:GFP)		This work
pGS70 (pUA66 P_{hisJ} WT:GFP)		This work
pGS5 (*HIS3-Pglk1-mTurquoise2-Tglk1)*		[17]
pMFM073 (*HIS3-Pglk1-mNeonGreen-Tglk1)*		[72]

Supplementary Table 3. Primers used in this study

Primers	Sequence 5'->3'	Description
GS_245	cagaatttgcatgccgtgac	*ΔargR:kan*^{R} cassette amplification from the keio strain, from the evolved lines and for Sanger sequencing
GS_246	ccttatgtattcattgtgtgaatgac	
GS_140	catcctgactagtctttcaggc	*hisG:kan*^{R} cassette amplification from the keio strain
GS_177	caaacttcgcgctgtattcc	
GS_281	cctttcgtcttcacctcgagacggcacctacgacaagatg	Primers used to amplify the hisJ promoter to construct the plasmid pUA66:P_{hisJ} :GFP reporters
GS_282	tctccttcttaaatctagaggatccttaaaccagagagagcgatagcac	
pUA_fw	ggatcctctagatttaagaaggaga	Amplification of the backbone from the pUA:GFP plasmid
pUA_rv	ctcgaggtgaagacgaaagg	
GS_259	aaatagagaagaacaagcaagatttttccctacccctattgggcatgccggtcgacggatctgatatcacc	*ecm21* KO cassette amplification for pH3FS
GS_260	attcattcttcatcactcatcaaaggcactatttcgtcataacgcggaggatggtgtcgacaacccttaat	
GS_261	gcattattctaatataacagttaggagaccaaaaagaaaaagaaatgtcagtcgacggatctgatatcacc	*gdh1* KO cassette amplification for pH3FS
GS_262	agactatttaaaatacatcaccttggtcaaacatagcatcagagaccttgatggtgtcgacaacccttaat	
GS_332	aagcatgccagtgttgaaatcagacaatttcgatccattggaagaagcttatggtgtcgacaacccttaat	*glt1* KO cassette amplification for pH3FS
GS_333	tgactagctaattctttcaatagtttgtaatcacgttgaacgataccaccgtcgacggatctgatatcacc	
GS_363	aatgacaagcgaaccagagtttcagcaggcttacgatgagatcgtttcttatggtgtcgacaacccttaat	*gdh3* KO cassette amplification for pH3FS
GS_364	agcgcttacggctaaaaaacgtctccctggtcaagcattgcgtcagccacgtcgacggatctgatatcacc	
GS_367	catacaaaacaaggatattaaattcacaacaataaaaagaataaagaatgatggtgtcgacaacccttaat	*gdh2* KO cassette amplification for pH3FS
GS_368	attgaagctcaagcacttgcctccgcttctctttttaacccaccaataaagtcgacggatctgatatcacc	
GS_288	cgatctggctgcaggacgtctggatgctgcgttacaagatgaagttgctggagcctgacatttatattcc	Amplification of the *neo-ccdB* cassette to replace the promoter region of *hisJ*
GS_289	atggcgtcaatcttcttcgcttttaaggacgggattaacgcatccagcgggtcccgctcagaagaactc	
