# Supplementary material for: Enhanced metabolic entanglement emerges during the evolution of an interkingdom microbial community
Source: Nat Commun. 2024 Aug 22;15:7238. doi: 10.1038/s41467-024-51702-1 (PMC11341674; doi:10.1038/s41467-024-51702-1)
Supplement: Supplementary file 3 — Description of Additional Supplementary Files [file 41467_2024_51702_MOESM3_ESM.pdf]

Description of Additional Supplementary Information

Supplementary Data 1: Recurrent mutations in *E. coli*

Supplementary Data 2: Recurrent mutations in *S. cerevisiae*

Supplementary Data 3: Complete mutation dataset obtained from breseq for *E. coli*

Supplementary Data 4: Complete mutation dataset obtained from breseq for *S. cerevisiae*

Supplementary Data 5: Original *E. coli* proteomics data presented in Supplementary Fig. 5

Supplementary Data 6: Original *S. cerevisiae* proteomics data from the same cocultures as shown in Supplementary Fig. 5

Supplementary Data 7: Original *S. cerevisiae* proteomics data for Fig. 2i and Supplementary Fig. 6

Supplementary Data 8: *E. coli* strains used in this study

Supplementary Data 9: *S. cerevisiae* strains used in this study
