# Supplementary material for: Enhanced metabolic entanglement emerges during the evolution of an interkingdom microbial community
Source: Nat Commun. 2024 Aug 22;15:7238. doi: 10.1038/s41467-024-51702-1 (PMC11341674; doi:10.1038/s41467-024-51702-1)
Supplement: Supplementary file 13 — Reporting Summary [file 41467_2024_51702_MOESM13_ESM.pdf]

nature portfolio

Corresponding author(s): Victor Sourjik

Last updated by author(s): Aug 4, 2024

Reporting Summary

Nature Portfolio wishes to improve the reproducibility of the work that we publish. This form provides structure for consistency and transparency in reporting. For further information on Nature Portfolio policies, see our Editorial Policies and the Editorial Policy Checklist.

Statistics

For all statistical analyses, confirm that the following items are present in the figure legend, table legend, main text, or Methods section.

n/a	Confirmed	
☐	☒	The exact sample size (*n*) for each experimental group/condition, given as a discrete number and unit of measurement
☐	☒	A statement on whether measurements were taken from distinct samples or whether the same sample was measured repeatedly
☐	☒	The statistical test(s) used AND whether they are one- or two-sided<br>*Only common tests should be described solely by name; describe more complex techniques in the Methods section.*
☒	☐	A description of all covariates tested
☒	☐	A description of any assumptions or corrections, such as tests of normality and adjustment for multiple comparisons
☐	☒	A full description of the statistical parameters including central tendency (e.g. means) or other basic estimates (e.g. regression coefficient) AND variation (e.g. standard deviation) or associated estimates of uncertainty (e.g. confidence intervals)
☐	☒	For null hypothesis testing, the test statistic (e.g. *F*, *t*, *r*) with confidence intervals, effect sizes, degrees of freedom and *P* value noted<br>*Give P values as exact values whenever suitable.*
☒	☐	For Bayesian analysis, information on the choice of priors and Markov chain Monte Carlo settings
☒	☐	For hierarchical and complex designs, identification of the appropriate level for tests and full reporting of outcomes
☒	☐	Estimates of effect sizes (e.g. Cohen's *d*, Pearson's *r*), indicating how they were calculated

Our web collection on statistics for biologists contains articles on many of the points above.

Software and code

Policy information about availability of computer code

Data collection	No customized software was used for data collection
Data analysis	Growth plots were generated with a custom-written Python script and quantification of growth parameters was done using QurvE (non-parametric model). Statistical analyses were performed with GraphPad Prism v9.0.2. Analysis of the sequencing data was performed using breseq and Integrative Genomics Viewer (IGV- version 2.8.9). Flow cytometry data were analyzed using FlowJo (BD Biosciences). Proteomic log-ratio and significance value (Student´s t-Test) calculation was performed as a basis for volcano plots with Perseus. Protein association network analysis and functional enrichment were performed with STRING. For metabolomics analysis, extracted ion chromatograms of the [M+H]+ forms were integrated using Tracefinder 5.2 software (Thermo Fisher Scientific)

For manuscripts utilizing custom algorithms or software that are central to the research but not yet described in published literature, software must be made available to editors and reviewers. We strongly encourage code deposition in a community repository (e.g. GitHub). See the Nature Portfolio guidelines for submitting code & software for further information.

Data

Policy information about availability of data

All manuscripts must include a data availability statement. This statement should provide the following information, where applicable:

- Accession codes, unique identifiers, or web links for publicly available datasets
- A description of any restrictions on data availability
- For clinical datasets or third party data, please ensure that the statement adheres to our policy

Data availability:
Original proteomics and sequencing data have been deposited in public repositories as indicated in Materials and Methods. Source data are provided as Source Data file. All materials are available from the corresponding author upon request. The proteomics data have been deposited to the ProteomeXchange Consortium via the PRIDE partner repository with the dataset identifier PXD047443 [http://www.ebi.ac.uk/pride/archive/projects/PXD047443]. Original fasta sequencing data are deposited in NCBI under the Bioproject PRJNA1049669 [https://www.ncbi.nlm.nih.gov/bioproject/PRJNA1049669] for CF50 and under the Bioproject PRJNA1051099 [https://www.ncbi.nlm.nih.gov/bioproject/PRJNA1051099] for CF269.

Research involving human participants, their data, or biological material

Policy information about studies with human participants or human data. See also policy information about sex, gender (identity/presentation), and sexual orientation and race, ethnicity and racism.

Reporting on sex and gender	no human participants, their data, or biological material was used
Reporting on race, ethnicity, or other socially relevant groupings	no human participants, their data, or biological material was used
Population characteristics	no human participants, their data, or biological material was used
Recruitment	no human participants, their data, or biological material was used
Ethics oversight	no human participants, their data, or biological material was used

Note that full information on the approval of the study protocol must also be provided in the manuscript.

Field-specific reporting

Please select the one below that is the best fit for your research. If you are not sure, read the appropriate sections before making your selection.

☒ Life sciences ☐ Behavioural & social sciences ☐ Ecological, evolutionary & environmental sciences

For a reference copy of the document with all sections, see nature.com/documents/nr-reporting-summary-flat.pdf

Life sciences study design

All studies must disclose on these points even when the disclosure is negative.

Sample size	No calculations were performed to determine the sample size. Sample size is indicated in each figure legend.
Data exclusions	No data were excluded
Replication	Experiments were performed with independent replicates as specified in figure legends
Randomization	Randomization was used during sample preparation
Blinding	No blinding wass used during sample preparation.Blinding was applied during data analysis

Reporting for specific materials, systems and methods

We require information from authors about some types of materials, experimental systems and methods used in many studies. Here, indicate whether each material, system or method listed is relevant to your study. If you are not sure if a list item applies to your research, read the appropriate section before selecting a response.

Materials & experimental systems

n/a	Involved in the study
☒	☐ Antibodies
☒	☐ Eukaryotic cell lines
☒	☐ Palaeontology and archaeology
☒	☐ Animals and other organisms
☒	☐ Clinical data
☒	☐ Dual use research of concern
☒	☐ Plants

Methods

n/a	Involved in the study
☒	☐ ChIP-seq
☐	☒ Flow cytometry
☒	☐ MRI-based neuroimaging

Plants

Seed stocks	no plant material was used
Novel plant genotypes	no plant material was used
Authentication	no plant material was used

Flow Cytometry

Plots

Confirm that:

☐ The axis labels state the marker and fluorochrome used (e.g. CD4-FITC).

☐ The axis scales are clearly visible. Include numbers along axes only for bottom left plot of group (a 'group' is an analysis of identical markers).

☐ All plots are contour plots with outliers or pseudocolor plots.

☒ A numerical value for number of cells or percentage (with statistics) is provided.

Methodology

Sample preparation	Co-cultures of organisms expressing different fluorescent markers were prepared as described in Materials and Methods.
Instrument	BD Fortessa Flow Cytometer
Software	Flow cytometry data were analyzed using FlowJo (BD Biosciences)
Cell population abundance	no sorting was performed
Gating strategy	The gating strategy used to distinguish yeast from bacteria, and the respective subpopulations, is described in Material and Methods and in ref. #17 and a figure exemplifying the gating strategy is reported in the supplementary material of that previous publication.

☐ Tick this box to confirm that a figure exemplifying the gating strategy is provided in the Supplementary Information.
